# Occlusion intestinale aiguë révélant une grossesse abdominale: à propos d'un cas

**DOI:** 10.11604/pamj.2018.31.155.17187

**Published:** 2018-10-31

**Authors:** Ibrahima Sitor Souleymane Sarr, Magatte Faye, Papa Mamadou Faye, Mamadou Seck, Ousmane Ka, Madieng Dieng

**Affiliations:** 1 Service de Chirurgie Générale, Centre Hospitalier et Universitaire Aristide Le Dantec de Dakar, Sénégal

**Keywords:** Grossesse abdominale, aménorrhées, traitement chirurgical, Abdominal pregnancy, amenorrhea, surgical treatment

## Abstract

La grossesse abdominale (GA) se définit comme la nidation et le développement de l'œuf fécondé dans la cavité péritonéale. Elle reste une variété rare de grossesse ectopique, dont la découverte peut être fortuite simulant une urgence chirurgicale. Nous rapportons un cas rare d'une occlusion intestinale aiguë fébrile révélant une grossesse abdominale chez une patiente âgée de 27ans sans antécédents pathologiques particuliers, reçue pour la prise en charge d'un syndrome occlusif évoluant depuis une semaine. L'examen physique retrouvait un abdomen distendu luisant, sensible dans son ensemble et météorisé. Le bilan biologique montrait des globules blancs à 20300, le taux d'hémoglobine à 7,2g/dL. L'exploration chirurgicale avait objectivé un hémopéritoine de 2000ml, une masse retro-utérine encapsulée, adhérent fortement au méso-sigmoïde et au sigmoïde responsable d'une sténose du colon descendant. Il a été réalisé une exérèse monobloc dont l'effraction montrait un placenta accolé au méso-sigmoïde relié au fœtus, une annexectomie droite, une colostomie.

## Abstract (en)

Abdominal pregnancy (AP) is defined as the implantation and the development of the fertilized egg in the peritoneal cavity. It is a rare type of ectopic pregnancy, whose detection can be fortuitous, mimicking surgical emergency. We report a rare case of acute febrile bowel obstruction revealing abdominal pregnancy in a 27-year old patient, with no particular past medical history, admitted to hospital for the treatment of occlusive syndrome evolving over the last week. Physical examination showed shiny, distended, meteoric, completely sensitive abdomen. Laboratory tests showed white blood cell count 20300, haemoglobin 7,2g/dL. Surgical exploration showed hemoperitoneum 2000ml, retrouterine encapsulated mass, strongly adherent to the mesosigmoid and to the sigmoid, responsible of a stenosis of the descending colon. The patient underwent monobloc exeresis, which showed a portion of placenta next to the mesosigmoid attached to the fetus, right annexectomy and colostomy.

## Introduction

La grossesse abdominale (GA) se définit comme la nidation et le développement de l'œuf fécondé dans la cavité péritonéale. Elle est le plus souvent secondaire, liée à un avortement tubo-abdominal ou à une rupture de grossesse extra-utérine (GEU) tubaire [1]. Elle reste une variété rare de grossesse ectopique, dont la découverte peut être fortuite simulant, dans certains cas une urgence chirurgicale abdominale. Le diagnostic pré-opératoire de GA est difficile, le caractère non spécifique des symptômes, le polymorphisme clinique lié au terme diagnostique [2]. Le traitement est essentiellement chirurgical. Elle est responsable d'une mortalité périnatale importante. Nous rapportons un cas rare d'une occlusion intestinale aiguë fébrile révélant une grossesse abdominale.

## Patient et observation

**Figure 1:**
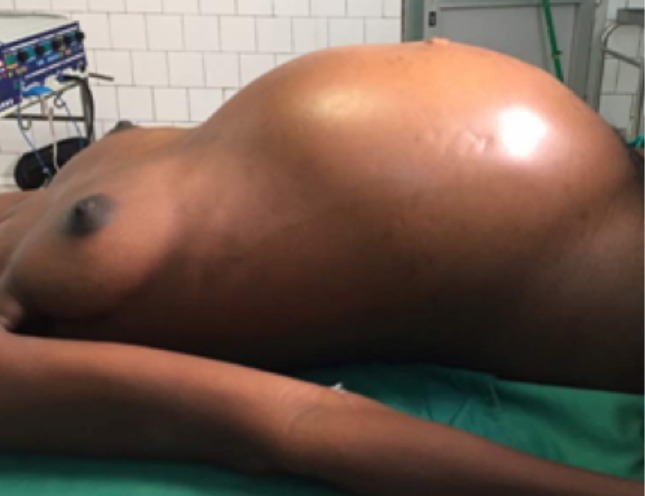
Vue pré-opératoire avec abdomen distendu luisant

**Figure 2:**
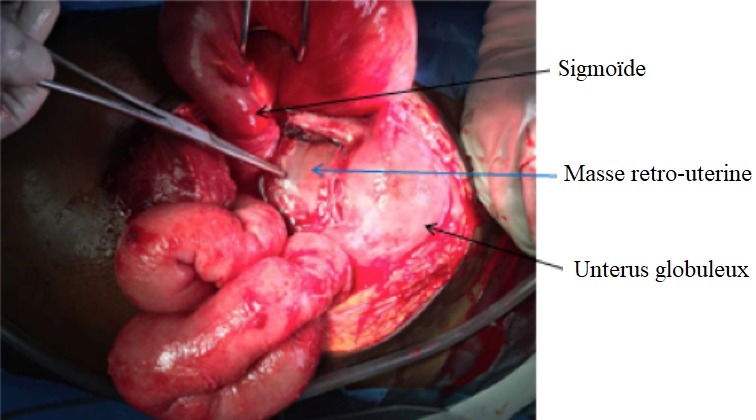
Vue opératoire masse rétro-utérine accolée au méso-sigmoïde

**Figure 3:**
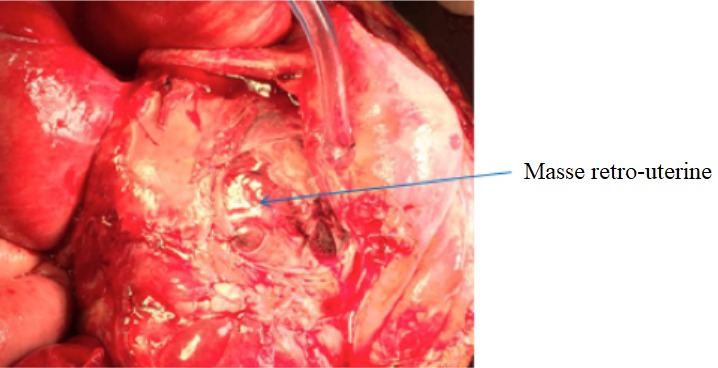
Vue opératoire masse rétro-utérine encapsulée

**Figure 4:**
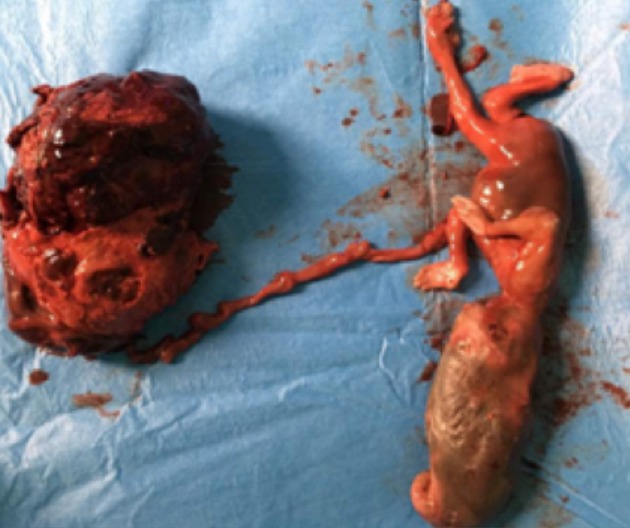
Pièce opératoire montrant le fœtus relié à son placenta

Il s'agit de FN patiente âgée de 27ans sans antécédents pathologiques particuliers, reçue le 25/08/2017 pour la prise en charge d'un syndrome occlusif fait de douleur abdominale, vomissement et arrêt des matières et des gaz évoluant depuis une semaine. L'interrogatoire révèle une notion de douleurs abdominales persistantes depuis 2 mois ayant nécessité son hospitalisation en structure périphérique avec traitement médical sans amélioration d'où sa référence après réalisation d'une échographie abdominale. L'examen à l'entrée retrouvait une patiente en mauvais état général, muqueuses pales anictériques. La température était à 38,8°c, le pouls 120bpm et la tension artérielle à 140/10. L'examen physique retrouvait un abdomen distendu luisante, sensible dans son ensemble et météorisé (Figure 1). Le toucher rectal retrouvait une ampoule rectale vide. Le bilan biologique réalisé montrait des globules blancs à 20300, le taux d'hémoglobine à 7,2g/dL et des plaquettes à 90,000 elts/mm³. L'échographie montrait un hémopéritoine de grande abondance et une grossesse abdominale non viable de 16 semaines d'aménorrhées. L'exploration chirurgicale avait objectivé un hémopéritoine de 2000ml, une masse retro-utérine encapsulée d'environ 20cm molle et friable, adhérent fortement au méso-sigmoïde et au sigmoïde responsable d'une sténose avec dilatation du colon descendant et transverse. La masse engaine l'appendice. L'utérus était de type gravide (Figure 2 et Figure 3). Comme gestes, il a été réalisé une exérèse monobloc de la masse dont l'effraction montrait un placenta accolé au méso-sigmoïde relié au fœtus d'environ 20cm par le cordon ombilical et des caillots d'environ 500ml, une annexectomie droite, une appendicectomie, une colostomie (Figure 4). Les suites opératoires se sont compliquées d'une éviscération septique à J7 sous-ombilicale de 3cm. Le rétablissement de la continuité digestive faite J60 avec des suites simples. L'examen anatomo-pathologique de la pièce confirmait la grossesse abdominale sans signe de malignité.

## Discussion

La grossesse abdominale (GA) se définit comme la nidation et le développement de l'œuf fécondé dans la cavité péritonéale. Il existe une forme secondaire, la plus fréquente, liée à un avortement tubo-abdominal ou à une rupture de grossesse extra-utérine (GEU) tubaire [1] et une forme primaire, rare, car devant satisfaire aux critères de Studdiford [3] qui sont les suivants: trompes et ovaires normaux, absence de fistule utéro péritonéale et contact exclusif de l'œuf avec la surface péritonéale. Chez notre patiente l'engainement de l'annexe droite permettait de retenir la forme secondaire. Elle reste une variété rare de grossesse ectopique [4]. Elle est responsable d'une mortalité périnatale comprise entre 40% et 95% [5]. On retrouve souvent les facteurs de risque de grossesse extra-utérine: stérilité, dispositif intra-utérin, antécédent de traumatisme utérin, interruption de grossesse par aspiration, cicatrice utérine, infections génitales [2]. Dans le cas rapporté, aucun facteur de risque n'a été trouvé. Le diagnostic pré-opératoire de GA est difficile, le caractère non spécifique des symptômes, le polymorphisme clinique lié au terme diagnostique [2]. Cependant, pour certains auteurs, il semble que l'existence de douleurs abdominales continues dans un contexte d'aménorrhée soit un signe majeur d'alerte. Parfois, la GA peut d'emblée se révéler par un hémopéritoine, une péritonite ou une occlusion intestinale [6]. Comme retrouvée chez notre patiente. Cette difficulté diagnostic s'associe au niveau socio-économique bas, la qualité du suivi de la grossesse en campagne. En effet la patiente étant primigeste, non instruite ignorait qu'elle était enceinte d'où l'intérêt d'une meilleure sensibilisation.

L'échographie est d'un apport capital devant le polymorphisme clinique de la GA. En effet, elle permet dans plus de 50% des cas de poser le diagnostic préopératoire devant un utérus vide, une absence de paroi utérine autour du fœtus qui est en contact direct avec les échos intestinaux [7]. Elle nous a permis de confirmer ce diagnostic chez notre patiente. Cependant l'IRM est d'apport un décisif de l'IRM dans l'établissement du diagnostic qui retrouve un utérus vide, un fœtus dans la cavité abdominale non circonscrit par du tissu myométrial et la localisation placentaire extra-utérine fournissant de précieux renseignements pré-opératoires [2]. Le traitement de la GA est presque toujours chirurgical. Cependant, le geste opératoire peut être différé au voisinage de la période de viabilité fœtale, à condition que la surveillance maternelle et fœtale soit renforcée [7]. Dans notre cas, l'absence de viabilité du fœtus et le tableau d'OIA avec signes de sepsis imposent la réalisation d'une laparotomie. La voie cœliochirurgicale est, selon les auteurs, réalisable en cas de grossesse de moins de 12 SA et d'implantation compatible avec un geste cœliochirurgicale [8]. La délivrance complète doit être l'idéale si elle est de réalisation aisée après inventaire des rapports du placenta avec des organes pelvi-abdominaux. Le placenta peut être laissé in situ après clampage du cordon au raz de la surface placentaire. La surveillance de sa résorption peut se faire par échographie doppler et ou par dosage des bêta-HCG plasmatiques [9]. Chez notre patiente l'implantation sur le méso-sigmoïde avec sténose du sigmoïde imposait la réalisation d'une exérèse et d'une colostomie du fait de l'incongruence de calibre, du saignement important et des conditions locales. Le diagnostic sera confirmé par l'examen histologique qui retrouve des implants trophoblastiques invasifs [10] comme ce fut le cas chez notre patiente.

## Conclusion

La grossesse abdominale (GA) se définit comme la nidation et le développement de l'œuf fécondé dans la cavité péritonéale. Elle reste une variété rare de grossesse ectopique. Le diagnostic pré-opératoire de GA est difficile par le caractère non spécifique des symptômes, le polymorphisme clinique dont l'occlusion intestinale est une des rares variétés. Le traitement de la GA est essentiellement chirurgical.

## Conflits d’intérêts

Les auteurs ne déclarent aucun conflit d’intérêts.
